# Visual-Tactile Spatial Multisensory Interaction in Adults With Autism and Schizophrenia

**DOI:** 10.3389/fpsyt.2020.578401

**Published:** 2020-10-23

**Authors:** Jean-Paul Noel, Michelle D. Failla, Jennifer M. Quinde-Zlibut, Zachary J. Williams, Madison Gerdes, John M. Tracy, Alisa R. Zoltowski, Jennifer H. Foss-Feig, Heathman Nichols, Kristan Armstrong, Stephan H. Heckers, Randolph R. Blake, Mark T. Wallace, Sohee Park, Carissa J. Cascio

**Affiliations:** ^1^Center for Neural Science, New York University, New York, NY, United States; ^2^Department of Psychiatry and Behavioral Sciences, Vanderbilt University Medical Center, Nashville, TN, United States; ^3^Vanderbilt Brain Institute, Vanderbilt University, Nashville, TN, United States; ^4^Department of Hearing and Speech Sciences, Vanderbilt University Medical Center, Nashville, TN, United States; ^5^Medical Scientist Training Program, Vanderbilt University School of Medicine, Nashville, TN, United States; ^6^School of Criminology and Justice Policty, Northeastern University, Boston, MA, United States; ^7^Core Scientific, Seattle, WA, United States; ^8^Department of Psychiatry and Seaver Center for Autism Research, Mount Sinai Hospital, New York, NY, United States; ^9^Department of Psychology, Vanderbilt University, Nashville, TN, United States; ^10^Vanderbilt Frist Center for Autism and Innovation, Nashville, TN, United States

**Keywords:** cross-modal congruency effect, peripersonal space, depth, logistic regression, psychopathology, somatic, developmental disorders, tactile perception

## Abstract

**Background:** Individuals with autism spectrum disorder (ASD) and schizophrenia (SZ) exhibit multisensory processing difficulties and social impairments, with growing evidence that the former contributes to the latter. However, this work has largely reported on separate cohorts, introducing method variance as a barrier to drawing broad conclusions across studies. Further, very few studies have addressed touch, resulting in sparse knowledge about how these two clinical groups may integrate somatic information with other senses.

**Methods:** In this study, we compared adults with ASD (*n* = 29), SZ (*n* = 24), and typical developmental histories (TD, *n* = 37) on two tasks requiring visual-tactile spatial multisensory processing. In the first task (crossmodal congruency), participants judged the location of a tactile stimulus in the presence or absence of simultaneous visual input that was either spatially congruent or incongruent, with poorer performance for incongruence an index of spatial multisensory interaction. In the second task, participants reacted to touch in the presence or absence of dynamic visual stimuli that appeared to approach or recede from the body. Within a certain radius around the body, defined as *peripersonal space (PPS)*, an approaching visual or auditory stimulus reliably speeds reaction times (RT) to touch; outside of this radius, in *extrapersonal space (EPS)*, there is no multisensory effect. PPS can be defined both by its size (radius) and slope (sharpness of the PPS-EPS boundary). Clinical measures were administered to explore relations with visual-tactile processing.

**Results:** Neither clinical group differed from controls on the crossmodal congruency task. The ASD group had significantly smaller and more sharply-defined PPSs compared to the other two groups. Small PPS size was related to social symptom severity across groups, but was largely driven by the TD group, without significant effects in either clinical group.

**Conclusions:** These results suggest that: (1) spatially static visual-tactile facilitation is intact in adults with ASD and SZ, (2) spatially dynamic visual-tactile facilitation impacting perception of the body boundary is affected in ASD but not SZ, and (3) body boundary perception is related to social-emotional function, but not in a way that maps on to clinical status.

## Introduction

Autism spectrum disorder (ASD) and schizophrenia (SZ) are clearly distinct clinical groups, but individuals on both the autism and schizophrenia spectra share some common categories of symptoms, including social and executive function deficits. There is evidence of considerable convergence in the nature and extent of these deficits (1–4), and common neural alterations in networks supporting social cognition (5–7). However, the phenotypic overlap in these high level social and cognitive domains is not complete (4, 8, 9), and more remains to be learned about points of divergence and convergence at multiple levels of function in these clinical groups (10). Given the dependence of higher level social cognitive functions on more basic component processes such as low-level perceptual integration, better characterization of sensory and perceptual function and their interrelationships in both groups could contribute to more complete understanding of both phenotypes.

In both ASD and SZ, sensory processing abnormalities are core to the phenotype, and difficulties in integrating and processing information across the different senses have been described [for a review, see (11)]. For example, individuals with ASD and individuals with SZ exhibit enlarged temporal multisensory binding windows, which reflect the temporal duration over which paired auditory and visual stimuli are bound together as a single percept (12–18). Among individuals with autism, this diminished temporal acuity for low-level multisensory stimuli is related to severity of social communication deficits (17, 19), and among patients with schizophrenia reduced temporal acuity is related to symptom severity with positive symptoms [i.e., hallucinations, delusions (15)]. These relationships prompt the idea that low-level multisensory processing may be a critical precursor to more complex, higher-order function. Indeed, aberrant temporal binding of audiovisual stimuli can have a profound impact on various aspects of language and social cognition, particularly speech comprehension (20), prosodic processing (21), and recognition of emotions in face/voice stimuli (22), all of which are impaired across both ASD and SZ (4, 23). While studies of multisensory binding have shown associations with social symptoms in ASD [e.g., socio-communicative abilities (17)], the association between multisensory processing and social function is less clear in SZ [e.g., (24)]. More vexingly, prior studies have been largely limited to the temporal domain (vs. spatial, for instance) and the pairing of audio and visual multisensory stimuli [(11) but see (2015) for a recent report indexing visuo-tactile interactions across both time and space in ASD].

Spatial multisensory integration is an inherent component in what is referred to as embodied cognition: the ability to separate oneself perceptually from the surrounding environment and to use that knowledge to plan and execute interactions within the environment. Recent work from our group and others has proposed that embodied cognition and its multisensory underpinnings may be a useful framework for comparing and contrasting the clinical profiles of autism and schizophrenia (25–27). For example, altered embodiment in ASD may cascade to influence deficits in non-verbal communication such as gesture (28) or violations of personal space (29). In SZ, altered embodiment could contribute to certain kinds of hallucinations (30). Converging inputs from touch, vision, and proprioception specify the location and boundary of the body within its environment, and the relative spatial properties of those inputs provide information about the potential for spatial interaction of the body with the social and physical environment. This spatial multisensory information is important in evaluating both how and when to enact motor programs in response to environmental events transpiring near or approaching one's body, and also the potential for threat or reward consequent to those interactions.

A commonly used paradigm to probe this spatial multisensory processing entails presentation of tactile stimulation together with auditory or visual stimuli manipulated to convey a sense of their approaching toward, or retreating away from, the body. By quantifying speeded reaction times (RTs) to approaching stimuli, one can define the individual's *peripersonal space (PPS)*, which is the radius immediately surrounding the body within which stimuli are perceived as physically relevant (31), whether for action or for self-defense (32). The boundary between PPS and extrapersonal space (EPS) is measured in terms of its size or radius and its sharpness or shallowness—the clarity of the delineation between peripersonal and extrapersonal space. PPS is highly malleable and can be modified by manual motor experience (31, 33), threat (34, 35), or social interaction. The interplay between social function and PPS is particularly noteworthy here, given our focus on individuals with ASD and SZ. In this regard, Teneggi et al. (36) demonstrated that in healthy controls PPS first shrinks upon encountering another individual, as to “give space,” and following a cooperative social interaction, it expands again, as if “sharing space.” Pellencin et al. (37) similarly demonstrated that the encoding of PPS is sensitive to the perceived morality of others. A prior study found evidence for smaller PPS in adults with autism using a audio-tactile paradigm (38), suggesting altered bodily self-consciousness in autism driven by differences in multisensory integration. In the present study, we used a similar, visuo-tactile paradigm in an effort to replicate and extend this finding of constricted PPS to adults with autism and compare to those with schizophrenia.

Previous research points to potentially opposite PPS profiles across ASD and SZ that may correspond to distinct elements of the clinical phenotype associated with each disorder. Specifically, individuals with ASD are less susceptible to the rubber hand illusion (39–41), a visual-tactile paradigm that manipulates the sense of body ownership, suggesting decreased influence of visual-tactile input on perceived body ownership, whereas individuals with schizophrenia are *more* susceptible (42, 43), suggesting *increased* visual-tactile influence on perception of body ownership. These divergent findings have been theorized to reflect the degree to which the two groups rely on external stimuli to update their body representation, with under-reliance on external input characterizing autism and over-reliance on external input characterizing schizophrenia (42). Based on these findings, we hypothesized similarly divergent peripersonal space profiles across groups, with individuals with ASD showing smaller PPSs with sharper borders and individuals with SZ showing larger PPSs with shallower borders (26). In an attempt to determine whether putative differences in PPS between ASD and SZ are specific, or may reflect more general effects of visual-tactile integration, we also administered the cross modal congruency task [CCE; (44)], where visual cues may facilitate or impaired tactile processing, but cues are always presented in the same location, near the body [see (45), for modulation of the CCE in the presence of others]. We did not have an a priori hypothesis for group differences in this task, given that there is reasonable grounds to predict both generalized and embodiment-specific differences in multisensory processing. In light of previous findings, we additionally hypothesized that differences in peripersonal space profiles would correlate with the severity of social deficits both within and across diagnostic groups.

## Methods

### Participants

A total of 84 participants took part in the current experiments. Participants were recruited into three groups: (1) adults with typical developmental histories (TD, *n* = 36), adults with autism (ASD, *n* = 26), and adults with schizophrenia (SZ, *n* = 22). Participants in all groups were between 18 and 60 years old (mean = 34.59, SD = 12.29). This age range is large and the average age across groups differed (the ASD cohort being younger; see Table 1). However, we considered this appropriate given difference in age of onset between autism and schizophrenia and our goal of assessing stabilized rather than first episode SZ patients. Importantly, age was incorporated as a covariate in analyses. Participants had no history of organic brain disease, lesions, head trauma, or neurological disorders, and were free from nerve damage, illnesses, or injuries that might influence sensation or perception in the tactile, visual, and auditory systems. All participants self-reported normal hearing and normal or corrected-to-normal vision (i.e., wore their prescription glasses). Recruitment was conducted through Vanderbilt University Medical Center clinical and research entities, including the Vanderbilt Kennedy Center, the Vanderbilt Early Psychosis Program, and community mental health providers and partners in the middle Tennessee area. Cognitive ability was measured using the 4-subtest Wechsler Abbreviated Scales of Intelligence—Second Edition [WASI-II (47)] and a full-scale estimated intelligence quotient (IQ) score of 70 or higher was required for inclusion in the study in all groups in order to assure that participants understood task demands. Further, similarly to age, cognitive ability was included as a covariate in all analyses.

**Table 1:** Sample and psychophysics paradigm subsample descriptive statistics.

	**ASD**	**SZ**	**TD**
Gender (M/F)	14/12	13/8 (1 unknown)	23/13
Mean Age (SD)	25.65 (6.05)	45.09 (9.94)	33.56 (11.19)
Handedness (%R, L, Other)	83%, 14%, 3%	87%, 13%, 0%	89%, 11%, 0%
Mean FSIQ (SD)	105.09 (17.54)	93.15 (17.07)	112.97 (13.31)
Mean SRS Total T-score (SD)	67.91 (12.63)	61.00 (11.74)	47.31 (7.67)
Mean ADOS calibrated severity score (SD)	7.59	N/A	N/A
Mean PANSS^a^ (positive)	N/A	15.21	N/A
Mean PANSS^a^ (negative)	N/A	15.54	N/A
**Medication**
Antipsychotic	–	*N* = 17	–
SSRI or SNRI	*N* = 3	*N* = 12	–
Mood stabilizer	–	*N* = 4	–
Benzodiazepine	*N* = 1	*N* = 2	–
Other	*N* = 2	*N* = 7	–
**Psychophysics**
Completed CCE task (% total sample)	23 (88.46%)	18 (81.81%)	33 (91.67%)
CCE excluded for < 10 trials/condition (% of those completing task)	3 (13%)	4 (22%)	3 (9%)
Completed PPS task (% total sample)	20 (76.92%)	22 (68.18%)	20 (50%)
PPS excluded for poor sigmoid fit (% of those completing task)	6 (30%)	7 (31%)	2 (10%)

a *From PANSS (n = 14) or converted from SAPS/SANS to PANSS (n = 8) using method of van Erp et al. (46)*.

Participants in the ASD and TD groups were free from any substance or alcohol abuse or dependence for at least 2 years prior to the study. The SZ group was also free from any substance or alcohol abuse or dependence, but this criterion was relaxed to the 3 months prior to the study and did not include nicotine, given the high rates of comorbidity between SZ and substance use disorders (48). Participants in the ASD and TD groups were free from antipsychotic medications and mood stabilizers, and medications with sedative effects, with the exception of one participant with ASD who reported taking a benzodiazepine. Participants in the TD group were additionally excluded for first degree relatives with either an ASD or SZ diagnosis, and personal history of any other psychiatric diagnosis (anxiety, mood disorders), ADHD, or learning disorders. Details of the entire sample, and the subsamples included in both psychophysical paradigms, are given in Table 1.

Diagnosis of autism was confirmed using research-reliable administration of the Autism Diagnostic Observation Schedule [ADOS-2 (49)], under the supervision of a licensed clinical psychologist. Diagnosis of schizophrenia was confirmed using diagnostic criteria in the Structured Clinical Interview-DSM-IV (SCID-IV); administered by a trained research assistant. Positive and negative SZ symptoms were assessed in the SZ group, either with the Scale for the Assessment of Positive Symptoms [SAPS (50)]/Scale for the Assessment of Negative Symptoms (SANS; 49, *n* = 8) or with the Positive and Negative Symptoms Scale [PANSS; (51), *n* = 14]. SAPS/SANS composite and global scores were converted to PANSS using linear regression as described in 51. Social symptom severity was quantified with the Social Responsiveness Scale adult self-report [SRS-2 (52)], which was administered to participants in all three groups. The SRS-2 is a 65 item measures that quantifies global traits relevant for ASD with a normalized total score as well as five clinical subscales (social awareness, social cognition, social communication, social motivation, and restricted/repetitive behavior). Higher total scores on the SRS-2 indicate greater social impairment. It has been validated in adults with ASD (53).

All participants gave their written informed consent prior to taking part in this study, which was approved by the Behavioral Sciences Committee at Vanderbilt University.

### Cross-Modal Congruency Effect (CCE)

**Figure 1:**
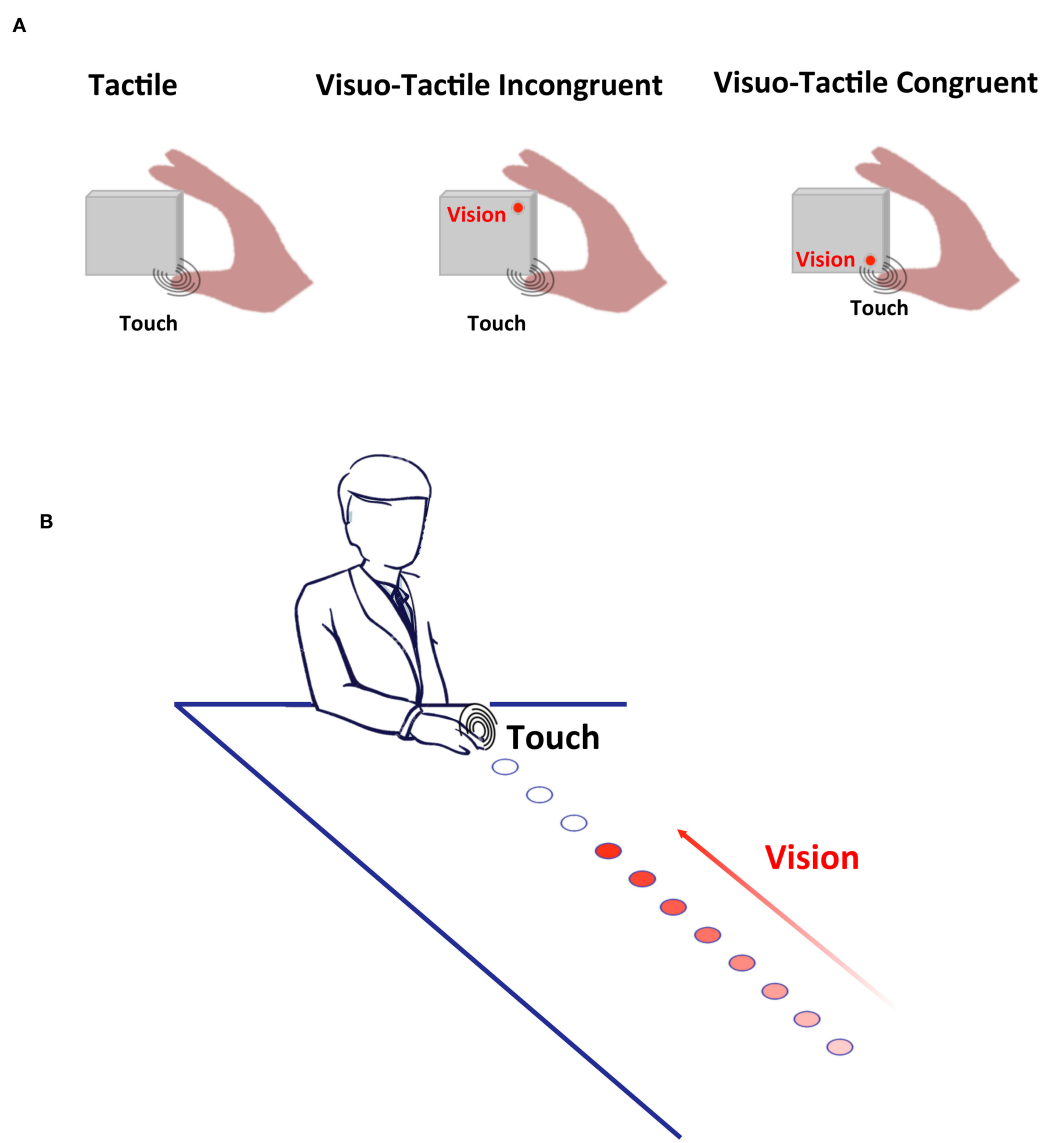
Experimental Methods. **(A)** Cross-Modal Congruency. Participants responded as fast as possible to touch being applied either to the thumb (depicted) or the index finger. On a fraction of trials, a visual stimulus was also presented, either incongruently with the location of touch (middle panel) or congruently with the location of touch (rightmost panel). **(B)** Peri-personal Space. On catch trials visual stimuli were presented alone, and on baseline trials, tactile stimuli were presented alone. On the experimental trials (depicted), touch was given when the train of visual stimuli terminated at different distances from the body, and either looming or receding from the participants. In this case looming stimuli is shown, with increasing intensity conveying the direction of movement of the light source.

Participants held in their right hand a purpose-made square block (8 × 8 × 6 cm) housing a pair of motors (Adafruit, New York, NY, 5V, 11,000 RPM, 0.9 g, 10 mm diameter, 2.7 mm thick) and LEDs (Adafruit, New York, NY, 4 mm × 9 mm, white). The block was held horizontally, the thumb and index fingers placed on top of the motors (Figure 1A). LEDs were immediately adjacent to their closest motor (congruent motor-LED pair), and 8 cm away from their incongruent motor. Motors and LEDs were all controlled via a micro-controller (Arduino Uno, Arduino, Somerville, MA, USA; 16 MHz). Visual stimuli had a duration of 10 ms, and vibrotactile stimulation lasted 100 ms. In line with prior studies, LED onset preceded tactile stimulation by 30 ms to counteract the instrinsic tendency for touch to be experienced as preceding visual stimulation when the two events occur simultaneously [see (44), for the original report using a similar setup and further see (54), for a characterization of the “principles of multisensory behavior” suggesting that the driver of multisensory RT facilitation is matching unisensory RTs, and not their physical simultaneity].

Participants made speeded discrimination responses regarding the position (thumb vs. index finger) to which the vibrotactile targets were presented, using a button press with the non-stimulated (left) hand. The vibrotactile stimulation was preceded by either no visual stimulation (a tactile-only baseline condition), a visual cue matching the location of the subsequent tactile target (congruent condition), or matching the location of the opposite finger (incongruent condition). In total 6 different trial types were possible (baseline, congruent, and incongruent for the two digits), and each unique condition was repeated 15 times, for a grand total of 90 trials. The inter-trial interval between tactile targets was between 1.5 and 2.5 s (uniform distribution), and trials timed out if there was no response within 10 s. This portion of the experiment took ~10 min, and was controlled via purpose-made MATLAB scripts (MathWorks, MA) communicating with the micro-controller via serial port.

RTs were calculated from the onset of vibrotactile stimulation. Responses slower than 2.0 s were discarded (<3% of all trials, no group difference). Data from participants with fewer than 10 trials per condition were excluded (*n* = 10; 3 ASD, 4 SZ, 3 TD). Responses that indicated tactile stimulation to the erroneous finger were marked as incorrect. Following the methods of Spence et al. (44), we subtracted values of RT and accuracy for congruent visuo-tactile stimulation from the incongruent condition in order to derive a measure of the impact of spatially congruency on low-level visuo-tactile RTs (44). Here we use these cross-modal congruency metrics (median RT and accuracy in percent) as outcome variables in separate ordinal logistic regression models (see *Analyses and Statistical Modeling* section below), with RTs and accuracy during the tactile-only (baseline) condition included as regressors in the corresponding models.

### Peri-Personal Space (PPS)

Participants comfortably rested their right hand on a custom-made box with a strip of LEDs (5 cm wide by 110 cm long) affixed to the top surface. LEDs were spaced in increments of 10 cm, starting at a distance of 5 cm from the edge of the box closest to the participant. In total there were 11 LEDs, one at each of the following distances: 5, 15, 25, 35, 45, 55, 65, 75, 85, 95, and 105 cm (Figure 1B). The visual component on each trial comprised sequential presentation of the LEDs, with presentation lasting 50 ms with an inter-stimulus interval of 200 ms between successive LEDs; this series of visual events conveyed the appearance of a single visual stimulus moving either toward the subject's hand (i.e., from D1 to D11; receding condition). A vibrotactile motor (Adafruit, New York, NY, 5V, 11,000 RPM, 0.9 g, 10 mm diameter, 2.7 mm thick) was attached to the participant's hand. Vibrotactile stimulation had a duration of 50 ms and could be activated in synchrony with one of the 11 different LEDs being turned on, or could be activated in isolation.

Participants were instructed to maintain gaze on a fixation point near the midpoint of the array of LEDs. They were informed that they would feel vibrotactile stimulation, and their task was to respond via button-press (with their non-stimulated, left hand) as fast as possible to this tactile stimulation. Additionally, they were informed that visual stimuli would be presented, but this visual input was task-irrelevant. The experiment comprised three type of trials; (1) experimental trials where tactile stimulation was given simultaneously with the onset of visual stimuli at a given distance and during a given movement direction (approaching or receding), (2) baseline trials were tactile stimuli was given in isolation at a timing that would have been equivalent to the visual stimuli being either at the closest or furthest location, and (3) catch trials wherein visual stimuli were presented either approaching or receding, but no tactile stimuli was given. In line with previous studies (55, 56), the rationale is that visual stimuli should enhance tactile processing when within but not when outside PPS. The facilitation ought to be most prominent when stimuli appear to be approaching the individual compared to when they appear to be receding (57). Baseline trials were measured in order to determine whether a multisensory effect is truly observed (visual-tactile RT < tactile-alone RT), and catch trials were introduced in order to limit an expectancy effect where tactile stimulation is more and more likely the longer it has been absent during visual stimulation (58). In this case, each of the 22 different experimental conditions (2 directions × 11 distances) were presented 16 times, each of the two baseline conditions (at temporal onset equivalent to D1 and D11) was presented 16 times, and each of the two catch conditions (approaching and receding) were presented 39 times [the report introducing this method to measure PPS, 54, counted with half the number of repetitions per experimental conditions (8), and 55, being the report with the largest number of individual subjects−164 subjects across 7 studies—similarly used 16 repetitions per experimental condition]. In total the experiment consisted of 462 repetitions (~17% catch), was divided in 3 blocks of equal length, and took ~40 min to complete. Inter-trial interval was set to 2.5 s.

Overall, participants were accurate at withholding responses during catch trials [<0.5% of trials, see e.g., (59)], and thus analysis was centered on RTs. Contrast between visuo-tactile RTs during approaching and receding motion (regardless of group) indicated that despite the inclusion of a number of catch trials, putative speeding in RT as a function of visuo-tactile proximity were contaminated by an expectancy effect; the longer the duration between trial onset and tactile stimulation, the faster the RTs (Supplementary Figure 1). To compensate for this effect and truly examine the impact of distance (and not time) on visuo-tactile RTs, we inverted the spatial dimension for the receding condition, and performed a subtraction equating time but differentiating distances. That is, D1 during approaching visual motion matches in time D11 during receding visual motion, D2 during approaching matches D10 during receding, and so forth. Hence, by performing this subtraction (e.g., approaching D1—receding D11) we eliminate the effect of time, and study exclusively the impact of distance (near vs. far), and direction (approaching vs. receding); the two aspects for which PPS neurons are selective (60). After performing the subtraction, in line with previous studies [e.g., (13)], we fit RTs to a sigmoidal function,

(1) $$\begin{array}{r} y\left(x\right)=\;\frac{y_{\min}+\;y_{\max}{\;}^{*}e^{\left(x-x_{c}\right)/b}}{1+\;e^{\left(x-x_{c}\right)/b}} \end{array}$$

where x represents the distance between visual and tactile stimuli, y(x) is the RT to touch at a given visual distance x, *y*_min_ and *y*_max_ are the saturation points of the sigmoidal which are fixed to the slowest and fastest mean RT in the experimental trials (i.e., not a free parameter), and *x*_*c*_ and *b* are, respectively, the central point and a parameter (negatively) proportional the slope of the sigmoidal at *x*_*c*_. These last two parameters are free parameters we fit to concisely describe PPS and represent its size (*x*_*c*_) and gradient (b)—how strongly are the near and far space separated. The parameters of subjects showing a good fit (a priori set to *R*^2^ > 0.5; TD = 18/20; ASD = 20/26; SZ = 15/22) were kept and contrasted across participants groups.

### Analyses and Statistical Modeling

We used a proportional odds logistic ordinal regression model for continuous data [i.e., a cumulative probability model with logit link (61, 62)] to assess the impact of distinct regressors on multisensory spatial processing. For the CCE task, we regressed the mean difference in RT during congruent and incongruent visuo-tactile stimulation, as well as the change in accuracy, on gender, age, full-scale IQ, baseline tactile performance, and diagnostic group. For the PPS task, we first summarized the pattern of RTs by an estimate of the size and gradient of PPS. These latter values were then submitted to a regression with age, gender, full-scale IQ, and diagnostic group as predictors. One individual in the schizophrenia group did not report their gender, and five individuals (3 ASD, 2 SZ) were missing full scale IQ scores; these missing values were handled using 40-fold multiple imputation as implemented by the *aregImpute* function in the R package *Hmisc* (63).

While SRS-2 scores indexing social symptoms were available for all participants, positive and negative symptom scales (SAPS and PANSS) were only available for the schizophrenia group. Thus, we examined Spearman correlations between these scales and the multisensory variables of interest separately from the regression models.

## Results

### Intact Cross-Modal Spatial Congruency Effect in ASD and SZ

**Figure 2:**
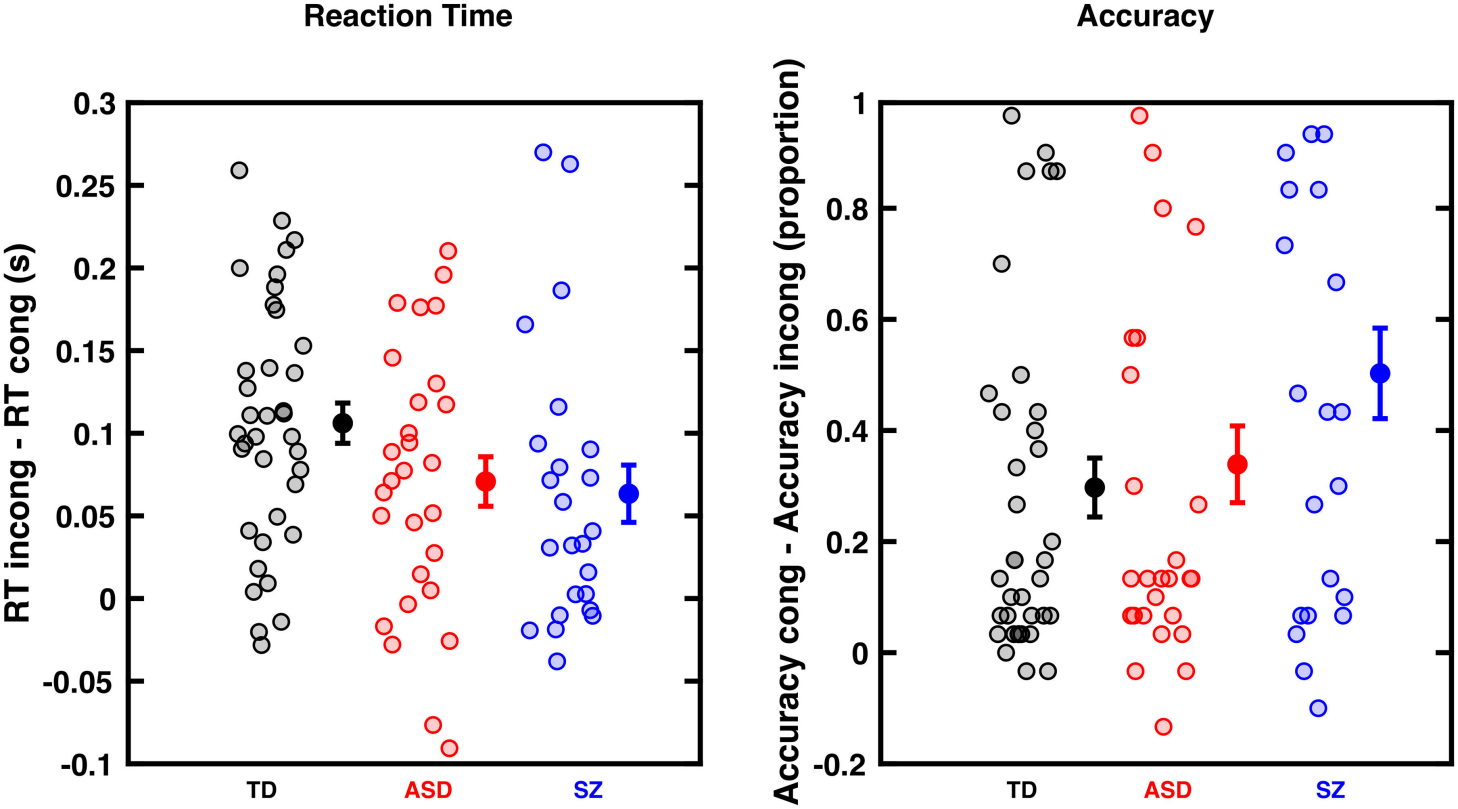
Cross-Modal Congruency Effect (CCE) for RTs (left) and accuracy (right) in typically developing (TD) individuals, as well as Autism Spectrum Disorder (ASD), and Schizophrenic (SZ) patients. **(A)** CCE is the difference between tactile RTs when preceded by an incongruent or congruent visual cue (measured in seconds). Controls (black) individuals show numerically a larger cross-modal congruency effect than ASD (red) and SZ (blue) individuals, but this difference is not statistically significant. **(B)** Similar format but subtracting accuracy in incongruent trials from accuracy in congruent trials. Groups did not statistically differ. Dots are individual participants, error bars show the mean and ±1 S.E.M.

As illustrated in Figure 2, all three groups of participants showed a cross-modal congruency effect, expressed both as a facilitation in RTs (Figure 2A, contrast to y = 0; all *p* < 0.0013) and enhanced response accuracy (Figure 2B, contrast to y = 0, all *p* < 3.5e-05) to tactile localization when a visual cue was spatially congruent as opposed to incongruent. The regression model assessing the influences on the cross-modal congruency effect as defined by RT suggested that none of the five predictors (diagnostic group, age, gender, IQ, and tactile-only RTs) predicted the multisensory congruency effect. The regression model assessing the impact of different predictors on the cross-modal congruency effect as defined by tactile localization accuracy suggested that baseline tactile accuracy in the absence of visual cues significantly predicted performance during the cross-modal congruency test (*aOR* = 0.87, CI_95_ [0.82, 0.91], *p* < 0.001), such that more accurate baseline tactile localization predicted less multisensory benefit regardless of age, gender, or diagnostic group.

### Peri-Personal Space Is Small and Its Boundary Sharp in ASD

**Figure 3:**
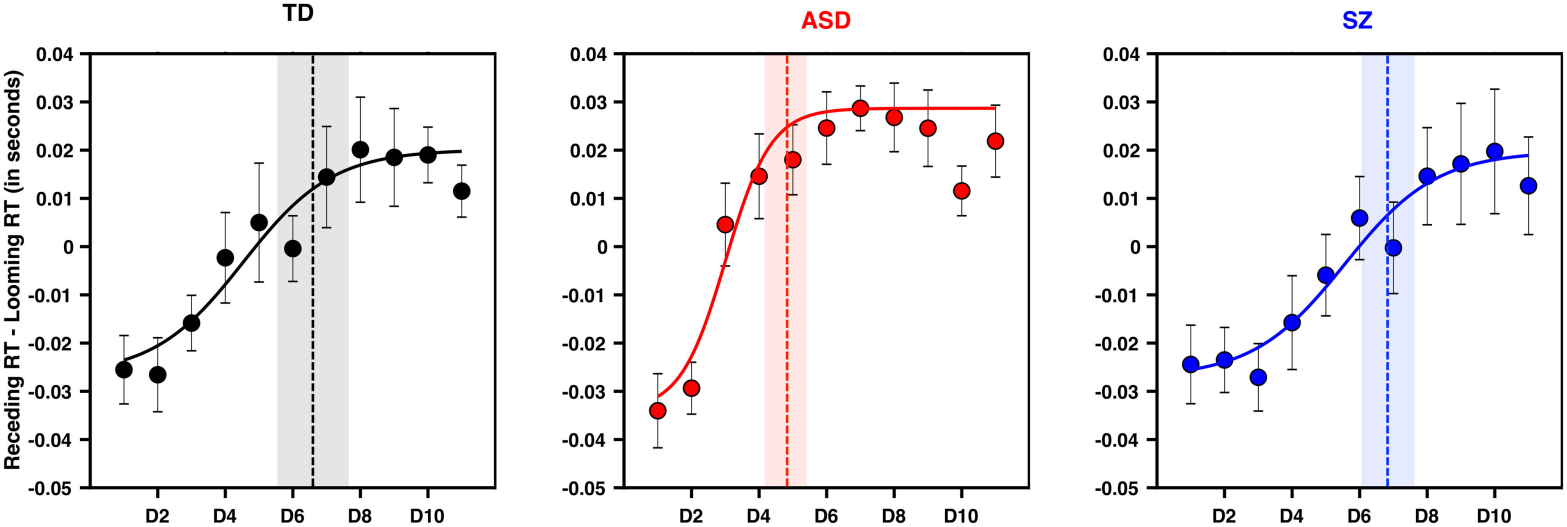
PPS is smaller and sharper in ASD than TD and SZ. Y-axis is the difference between tactile RTs during approaching and receding visual stimuli when matched for time. X-axis is distance, in the frame of reference of the approaching stimuli. Namely, D1 corresponds to D1 for approaching, and D11 for receding (which are matched in time). Negative values along the y-axis indicate a further facilitation as a function of distance for approaching than for receding visual stimuli, as would be expected of a PPS effect. Error bars are ±1 S.E.M., the vertical line in each panel (TD = black, ASD = red, SZ = blue) is the average central point of the sigmoidal fit (the fit shown is for the average subject). Shaded area is ±1 S.E.M.

After matching the temporal components of the PPS task and contrasting looming vs. receding visual stimuli in regard to enhancement of tactile RTs (see Methods section), all groups showed a profile of RTs suggesting tactile processing facilitation during multisensory trials at the nearest distance (Figure 3; expectancy-corrected multisensory RTs vs. unisensory tactile, all *ps* < 0.005). In line with prior studies [e.g., (55)] to succintly summarize the PPS data, we fit RTs to a sigmoidal function describing the size and sharpness (i.e., slope of the gradient) of the PPS boundary. These parameters were then submitted to statistical modeling, as described in the Methods section.

The model attempting to regress the size of PPS on diagnostic group, age, IQ, and gender suggested that only ASD as a diagnostic group significantly predicted PPS size such that a diagnosis of ASD was predictive of a smaller PPS (*aOR* = 0.09, CI_95_[0.02, 0.41], *p* = 0.002; see Figure 3). Schizophrenia as a diagnostic group was not a significant predictor of PPS size (*aOR* = 0.84, CI_95_[0.21, 3.45]; *p* = 0.814). A similar model assessing the gradient of boundary between PPS and EPS suggested that ASD as a diagnostic group significantly predicted a sharper PPS gradient (*a*OR = 0.18, CI_95_[0.04, 0.74], *p* = .0175). In contrast, a diagnosis of SZ did not hold significant predictive power as a determinant of PPS gradient (*a*OR = 1.4, CI_95_[0.35, 5.67], *p*=0.6344). Neither age, IQ, nor gender significantly predicted PPS size or gradient.

### Social Impairment Associated With Smaller PPS Across Groups, but Not Within Clinical Groups

**Figure 4:**
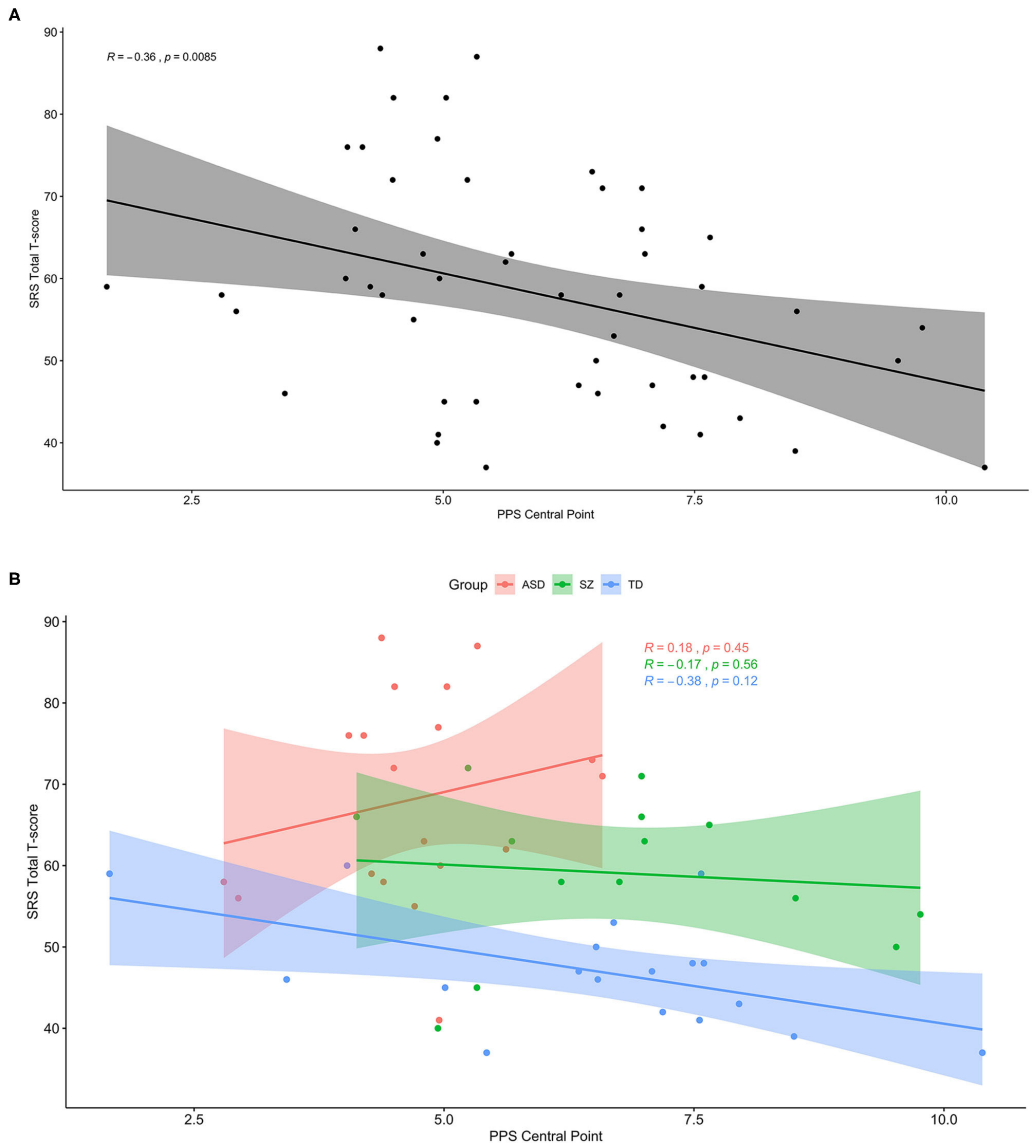
Smaller PPS is associated with more social-communication deficit, but only in the subclinical range. **(A)** Across the whole sample, greater social-communication deficits as measured by the total T score of the SRS-2 was associated with smaller peripersonal space (PPS). **(B)** The same plot with groups separated by color (ASD: red, SZ: green, TD: blue), showing a trendline similar to that for the whole sample only within the TD group.

In a secondary analysis, we used Spearman's correlation to determine the association between peripersonal space size and gradient with a measure of social-communication dysfunction, the total T score of the SRS-2. Although smaller PPS size was significantly associated with more severe social impairment in the whole sample (*r* = −0.36, *p* = 0.009), this association remained significant after Bonferroni correction and appeared driven by a non-significant trend in the TD group, and there were no significant associations in either clinical group (TD: *r* = −0.38*, p* = 0.12; ASD: *r* = 0.18, *p* = 0.45; SZ: *r* = −0.17, *p* = 0.56). PPS gradient was not associated with SRS scores either across or within groups. This secondary analysis is summarized in Figure 4.

### Schizophrenia Symptoms Do Not Significantly Correlate With PPS Size or Slope

Despite the lack of group effects for our SZ sample, based on findings from previous studies (64, 65), we conducted an exploratory analysis testing for an association between PPS size or gradient and symptoms of schizophrenia. We hypothesized that PPS size and/or its slope may relate to schizophrenia symptoms since positive symptoms have been linked with disturbances in the sense of self (66). However, we found no such association between PPS variables and positive (all *rs* < |0.35|, all *ps* > 0.17) or negative symptoms of SZ (all *rs* < |0.23|, all *ps* > 0.37).

## Discussion

A growing literature has emphasized deficits in sensory processing in ASD and SZ. Much of the work in characterizing these anomalies has been focused on multisensory processing, specifically examining the tolerance of these groups to temporal asynchronies between disparate signals arising within different sensory modalities. In addition to their temporal offset, however, another key feature ultimately leading to either the integration or segregation of sensory signals is their spatial disparity. This spatial factor has been less thoroughly explored within ASD and SZ. The present findings provide a start toward redressing this gap in knowledge by suggesting that that the spatial range over which visual stimuli facilitate tactile processing is diminished and has a more abrupt boundary relative to controls in ASD but not in SZ (peri-personal space experiment). However, visual-tactile integration in a more spatially constrained paradigm (cross-modal congruency experiment) was unaffected in both clinical groups, for whom spatially congruent visual stimuli facilitated tactile RTs similarly to that in the TD group. Broadly, these findings are consistent with recent observations from Poole et al. (67), in that they imply that the basic principles governing multisensory integration in ASD and controls is similar, but the exact spatial range over which interactions occur likely differ. While previous studies have reported associations between multisensory processing in the temporal domain and clinical symptoms in both groups (11, 19), we were unable to detect these associations for social symptoms, at least as indexed by the SRS-2. One consideration is that our sample was only assessed using the self-report version of the SRS-2, which depends on insight that may be diminished in both clinical groups.

The finding suggesting a sharper, more constricted PPS within the ASD group is in line with our predictions (26) derived from the observation that individuals with ASD are less susceptible to the rubber hand (39–41) and full-body (38) illusions than controls. Further, they corroborate and extend recent results from Mul et al. (38) by showing that whether PPS is mapped via an audio-tactile (38) or visuo-tactile (current study) pairing, PPS is more constricted and sharper in ASD. On the other hand, the data in SZ patients do not support our prediction (26), based on their heightened susceptibility to bodily illusions, of a larger PPS with a shallower border. Similarly, our results do not intuitively align with the replicated observation that patients with SZ need a relatively larger personal space (68, 69), nor do they replicate results from Di Cosmo et al. (70) that suggested individuals with SZ have a smaller PPS than controls when mapped via an audio-tactile pairing. Speculatively, it is possible that the sensory modality employed to index PPS—vision here and auditory in Di Cosmo et al. (70)— may explain the contradiction between the two studies, particularly given the much higher prevalence of auditory than visual hallucinations in SZ (71). Together, the findings highlight that while there are clear relations between aspects of embodiment [e.g., PPS (72)] and social aspects of personal space (35, 73), these relations are complex as they relate to clinical disorders with social deficits at their core.

The lack of an effect on the PPS task for our SZ group does not lend support to the theory advanced by Crespi and Dinsdale (25) that autism and schizophrenia represent diametrically opposed disorders of embodiment. However, the version of the task we used is non-social in nature (using only LEDs and vibrotactile stimuli); it is possible that with more social context in the stimuli (e.g., a ball being thrown), stronger group effects may have emerged. The theory of opposing embodiment was predicated on evidence from the rubber hand illusion, for which ASD and SZ patients, respectively, show reduced and enhanced susceptibility (39, 41, 43, 74). The rubber hand illusion is arguably a more interpersonal paradigm, for which the peak effect transfers the sense of one's bodily ownership to the representation of another body, or part of a body. With the PPS paradigm, on the other hand, this kind of exchange is not measured. Rather, what is measured is the radius surrounding the body in which external sensory events are perceived to have physical relevance, a much broader and less inherently social construct. The presence of a difference in PPS representation for ASD but not SZ may be consistent with a broader base of evidence for generalized multisensory integration differences in ASD relative to SZ (75–77). It would also be interesting to explore these questions in unmedicated, first-episode SZ patients who would presumably have more active positive symptoms than our cohort.

Despite the more constricted, more sharply defined PPS in adults with ASD, we did not find clear associations with clinical symptoms—either core autism or core schizophrenia symptoms in our ASD or SZ groups. However, in the whole sample, smaller PPS size was significantly associated with more social-communication impairment as measured by the SRS-2 total score. The SRS measure is considered a continuous trait index that can meaningfully span the general population and clinical groups (78, 79); however, in our sample, there was a clear difference across groups in how this index mapped onto PPS size. The global finding was influenced heavily by the association in the TD group, while those adults with ASD or SZ, particularly those with higher SRS-2 scores approaching the clinically significant range (above 60) did not show a clear relationship between PPS size and social-communication impairment. This raises the possibility of non-linear relations between social function and PPS across the full range of social-communicative function, in which milder symptoms align with predictions based on previous experiments in TD individuals, but more severe symptoms have a different, or possibly absent relation to PPS. It is also worth considering that self-reported symptoms in the clinical groups may suffer from low validity given limited insight, which would obscure potential correlations. The malleability of PPS in the presence of social (36) and threatening (34) external stimuli highlights the fact that PPS can be construed as a “state” measure, which may not correspond to more stable “traits” of social deficits [see (80), for evidence that PPS remaps even on the time-scale of seconds]. Supporting the idea that PPS and the rubber hand illusion are measuring more generalized and more socially-specific aspects of embodiment, respectively, most studies have reported clear associations between altered rubber hand illusion effects and clinical symptoms (39, 43, 74). Thus, future studies may opt for more socially-relevant visual stimuli in PPS paradigms (e.g., a ball being thrown toward the participant) to determine whether the expected relationships emerge in more social contexts.

All experimental groups—control, ASD, and SZ—showed a cross-modal congruency effect (44) of equal magnitude. Additionally, all three groups showed a PPS representation: reaction times to touch were modified by the spatial location of the visual stimulus. As such, the commonalities in multisensory processing between these groups outweighs their differences, despite the smaller size and sharper gradient of PPS in ASD. This complement of multisensory similarities and differences across groups may be interrogated in future work alongside previously-developed neural network models for PPS (80, 81). This is recommended as an approach that may help bridge from behavioral sensory deficits to putative neural circuitry anomalies relevant for multisensory integration.

The current study has a number of strengths, including the direct comparison of adults with schizophrenia and autism on a multisensory paradigm, the incorporation of spatial measures to complement the numerous studies that have focused on temporal processing, and the inclusion of two well-established visual-tactile interaction paradigms. This study also has some important limitations to consider. The sample sizes are modest, and there was some data loss for the PPS task due to attrition from the study and RTs that could not be fit to a sigmoid function. This data loss may have limited our ability to detect correlations with clinical symptoms. Differential use of medications across groups is an additional limitation that should also be considered, and, relatedly, our SZ cohort was chronic, stabilized, and thus perhaps representative of only one phase of the disease process. Future studies might include first-episode or prodromal patients to address this. Finally, our study was cross-sectional. Peripersonal space representation can be measured shortly after birth (82) and may form the basis of an emerging sense of self in infancy and early toddlerhood (83), the period in which autism symptoms are first evident. Thus, prospective longitudinal studies of this phenomenon and related tests of bodily self-consciousness in infants at high genetic risk for autism or other neuropsychiatric conditions may shed important light on whether and how the development of the sense of self goes awry in these populations.

## Data Availability Statement

The datasets presented in this study can be found in online repositories: https://osf.io/en3x8/.

## Ethics Statement

The studies involving human participants were reviewed and approved by Vanderbilt University Human Subjects Research Protection Program. The patients/participants provided their written informed consent to participate in this study.

## Author Contributions

CC, J-PN, MW, SP, and RB conceived and designed the study, interpreted the results, and drafted the manuscript. CC oversaw data collection an analyses. J-PN, AZ, JQ-Z, ZW, and MF contributed to data cleaning and analyses. HN, KA, MG, and JT collected the data. SH assisted with coordination of data collection, interpreted results, and drafted the manuscript. JF-F interpreted results and drafted the manuscript. All authors contributed to the article and approved the submitted version.

## Conflict of Interest

The authors declare that the research was conducted in the absence of any commercial or financial relationships that could be construed as a potential conflict of interest.
