# Supplementary material for: Visual-Tactile Spatial Multisensory Interaction in Adults With Autism and Schizophrenia
Source: Front Psychiatry. 2020 Oct 23;11:578401. doi: 10.3389/fpsyt.2020.578401 (PMC7644602; doi:10.3389/fpsyt.2020.578401)
Supplement: Supplementary file 1 [file Data_Sheet_1.docx]

**Supplementary Materials**

**Figure S1. Tactile RT as a function of visual stimuli location (x-axis) and motion direction (solid black = approaching; dashed gray = receding).** RTs during the presence of a visual stimulus were faster than tactile detection RTs alone (dashed black line at D1 and D11). However, the facilitation in detection appears largely driven by an expectation effect with both the approaching and receding visual stimuli eliciting their fastest RTs at large temporal intervals from stimulus onset. Error bars are +/- 1 S.E.M., data here are amalgamated across all participant groups.

**Figure S2.** **RTs and Accuracy in speeded tactile localization task.** RT (top) and accuracy (bottom) are plotted as a function of participant group (control, ASD, and SZ, respectively in black, red, and blue), and nature of preceding visual cue; none (baseline condition), congruent, or incongruent location (respectively, leftmost, center, and rightmost). Dots are individual participants, error bars show the mean and +/- 1 S.E.M.
